# Development and internal-external validation of a machine learning-based risk prediction model for multidrug resistance in mechanically ventilated ICU patients with chronic obstructive pulmonary disease

**DOI:** 10.3389/fmed.2026.1899252

**Published:** 2026-08-20

**Authors:** Weimin Xu, Yu Gu, Jing Xu, Yan Sun, Jiang Xin, Minxuan Ma

**Affiliations:** 1 Department of Clinical Laboratory, Affiliated Hospital of Jiangsu University, Zhenjiang, Jiangsu, China; 2 Department of Clinical Pharmacy, Baoying People’s Hospital, Baoying Clinical Medical College of Yangzhou University, Yangzhou, Jiangsu, China; 3 Department of Hospital-Acquired Infection Control, Affiliated Hospital of Jiangsu University, Zhenjiang, Jiangsu, China

**Keywords:** chronic obstructive pulmonary disease, machine learning, mechanical ventilation, multidrug resistance, nomogram, prediction model

## Abstract

**Objectives:**

This study aimed to develop and conduct internal-external validation of a machine learning-based risk prediction model for multidrug-resistant (MDR) infection in mechanically ventilated intensive care unit (ICU) patients with chronic obstructive pulmonary disease (COPD), so as to provide evidence for early identification of high-risk individuals, optimization of antimicrobial strategies, and reduction of infection-related adverse outcomes.

**Methods:**

We retrospectively analyzed 477 mechanically ventilated COPD ICU patients from the MIMIC-IV database, which were divided into a training set (333 cases) and an internal validation set (144 cases) at a ratio of 7:3, with MDR infection as the endpoint. Feature selection was performed using the Boruta algorithm and least absolute shrinkage and selection operator (LASSO). Seven machine learning algorithms were adopted for model training and construction with 10-fold cross-validation. Model performance was evaluated by AUC, accuracy, sensitivity, specificity, F1 score, calibration curve and decision curve analysis. External validation was conducted using 371 independent single-center clinical cases. The optimal model was interpreted by Shapley additive explanations (SHAP) analysis, and a predictive nomogram was established.

**Results:**

Five core predictors were identified, including red blood cell count, hemoglobin, hematocrit, blood urea nitrogen and creatinine. The K-nearest neighbor (KNN) model outperformed other algorithms, with an internal validation AUC of 0.642 (95% CI: 0.518-0.766) and an external validation AUC of 0.639 (95% CI: 0.508-0.769), accompanied by favorable accuracy, calibration and clinical net benefit. SHAP analysis indicated that hematocrit was the primary predictive factor; elevated red blood cell-related indexes increased MDR risk, while elevated blood urea nitrogen and creatinine decreased the risk.

**Conclusion:**

MDR infection in mechanically ventilated COPD ICU patients is closely associated with five routine laboratory indicators. The established KNN model and nomogram show favorable predictive value and generalizability, which can achieve rapid individualized risk quantification and serve as an auxiliary tool for early risk stratification and rational antimicrobial selection. It should be emphasized that this model is only used for preliminary screening and cannot replace microbial culture and drug susceptibility testing as the gold standard for definitive diagnosis.

## 1 Introduction

Chronic obstructive pulmonary disease (COPD) is a common, preventable, and treatable disease characterized by persistent airflow limitation. This airflow limitation is usually progressive and is associated with an abnormal chronic inflammatory response of the airways and lung tissues to noxious gases or particles, such as cigarette smoke (1). Patients with COPD have long-term airway structural damage and compromised immune function, making them susceptible to recurrent infections. When the disease progresses to moderate or severe stages, these patients often require transfer to the intensive care unit (ICU) and mechanical ventilation support (including invasive and noninvasive ventilation) to improve oxygenation and alleviate respiratory failure symptoms (2).

According to estimates by the World Health Organization (WHO), COPD ranks fourth in the global burden of disease and is projected to rise to third place by 2030 (3). In China, the prevalence of COPD among individuals aged 40 years and above has reached 13.7%, with nearly 100 million patients affected (4). Mechanical ventilation serves as an important therapeutic modality for ICU patients with COPD complicated by severe respiratory failure. However, as an invasive or semi-invasive procedure, it disrupts respiratory barrier function and increases the risk of hospital-acquired infections, among which multidrug-resistant (MDR) infections are particularly prominent (5). MDR infection was defined as bacterial resistance to three or more classes of antimicrobial agents with different mechanisms of action. These infections are difficult to treat, have limited antimicrobial options, significantly prolong the duration of mechanical ventilation and length of hospital stay, increase medical costs, and even lead to patient death, with mortality rates reaching 20%–50% (6).

Currently, the prediction of MDR infection in ICU patients with COPD receiving mechanical ventilation mainly relies on clinicians’ empirical judgment and single laboratory indicators. The lack of objective and accurate prediction tools often leads to antimicrobial misuse or delayed administration, further exacerbating the dissemination of drug resistance and adverse patient outcomes (7). Therefore, early and accurate prediction of MDR infection risk in this patient population holds significant clinical importance for optimizing clinical intervention strategies, rationalizing antimicrobial use, reducing the spread of resistant organisms, and improving patient prognosis (8). With the rapid development of information technology and data mining techniques, machine learning has been increasingly applied in the field of medical infection risk prediction. It could integrate multidimensional clinical indicators to construct objective and efficient prediction models, providing novel approaches for early warning of MDR infections (9).

This study was based on the MIMIC-IV database and single-center clinical data. Multiple machine learning algorithms have been employed to construct a risk prediction model for MDR infection in ICU patients with COPD receiving mechanical ventilation. Model performance was evaluated through internal and external validation. SHAP analysis was combined with other methods to elucidate the mechanisms of predictive features, and a nomogram was developed to achieve individualized risk quantification, thereby providing scientific evidence for the precise prevention and control of MDR infections in this clinical population.

## 2 Materials and methods

### 2.1 Data sources

The data used in this study were obtained from MIMIC-IV 3.0 (Medical Information Mart for Intensive Care) and single-center clinical data. The MIMIC-IV is a large, publicly available critical care research database that includes demographic information, commonly used laboratory indicators upon admission, comorbidities, medication records, and survival outcomes for ICU patients. One member of the research team was trained and certified (certificate number: 68821009) and was responsible for database access and data extraction. The MIMIC database followed the review board protocol, and all patient personal information was deidentified via random codes to identify specific patients. The external validation set data were derived from a single-center clinical ICU in Affiliated Hospital of Jiangsu University, and the ethics committee of this center approved the study (ethical approval number: KY2026K0509).

### 2.2 Inclusion and exclusion criteria

The inclusion criteria were as follows: (1) patients diagnosed with COPD who received mechanical ventilation (including invasive and noninvasive ventilation) for ≥24 h in the intensive care unit (ICU), identified via International Classification of Diseases (ICD-9 and ICD-10) codes; (2) aged ≥ 18 years; (3) first admitted to the ICU; and (4) completed relevant laboratory examinations during hospitalization with complete clinical documentation.

The exclusion criteria were as follows: (1) definite diagnosis of MDR infection prior to admission; (2) total intensive care unit (ICU) stay <24 h; (3) excessive missing laboratory indicators and other clinical information (missing rate > 5%); and (4) comorbid malignancy, severe immunodeficiency, or other severe infectious diseases (such as active pulmonary tuberculosis or fungal pneumonia). On the basis of these inclusion and exclusion criteria, a total of 477 patients diagnosed with COPD who received mechanical ventilation in the ICU from the MIMIC database were enrolled for analysis (used for the training set and internal validation set). Additionally, 371 patients from a single-center ICU were included in the external validation set.

### 2.3 Data extraction

The research team extracted and managed the data via structured query language (SQL) and PostgreSQL 15. The extracted data included demographic information (age, sex, body mass index, etc.), laboratory indicators (red blood cell count, hemoglobin, hematocrit, blood urea nitrogen, creatinine, etc.), comorbidities, and outcomes (whether MDR infection occurred) for ICU patients. MDR bacteria were defined as pathogenic bacteria resistant to ≥3 classes of antimicrobial agents with different mechanisms of action. The extraction indicators for the external validation set were consistent with those for the training set and internal validation set, including age, sex, red blood cell count (RBC), hemoglobin (HGB), hematocrit (HCT), blood urea nitrogen (BUN), creatinine (CREA), and MDR infection outcome for ICU patients.

### 2.4 Feature selection

The Boruta algorithm and LASSO regression were jointly employed for feature variable selection. Priority was given to retaining indicators with strong clinical significance, high model contribution, and no redundancy. Notably, clinically recognized risk factors including mechanical ventilation duration, pre-ICU antibiotic exposure history, tracheotomy status, and glucocorticoid use were not included in the final model. The primary reason is that the above variables had high missing rates and inconsistent recording standards in the single-center external validation cohort, which failed to meet the data quality requirements for stable modeling. To ensure the consistency of variables between the internal and external cohorts and the reliability of model generalization, we finally only included routine laboratory indicators with complete records and unified definitions. This data availability limitation may restrict the discriminatory performance of the model to a certain extent. Five core predictors were ultimately identified, namely, red blood cell (RBC) count, hemoglobin, hematocrit, blood urea nitrogen (BUN), and creatinine, which were used for subsequent model construction.

### 2.5 Model development

The 477 ICU patients with COPD who were receiving mechanical ventilation were divided into a training set (333 patients) and an internal validation set (144 patients) at a 7:3 ratio. Seven machine learning algorithms were used to construct prediction models in the training set: XGBoost, LightGBM, CatBoost, SVM, KNN, GNB, and NN. A total of 10-fold cross-validation was adopted for parameter optimization, and multiple resampling strategies including synthetic minority oversampling technique (SMOTE), ADASYN, and random undersampling were applied to address sample imbalance, with a comprehensive multi-dimensional comparison of model performance under different strategies, ensuring the stability and accuracy of model training.

### 2.6 Model evaluation

Model performance was evaluated in three dimensions: discrimination, calibration, and clinical utility. Discrimination was assessed via the area under the receiver operating characteristic curve (AUC), accuracy, sensitivity, specificity, and F1 score. Calibration was comprehensively evaluated via three complementary approaches: visual calibration curves, Brier score, and the Hosmer-Lemeshow (HL) goodness-of-fit test for binary endpoints; a smaller Brier score and HL test *P*-value > 0.05 jointly indicate better agreement between the predicted probabilities and actual observed probabilities. Clinical utility was assessed via decision curve analysis (DCA), which compared the net benefit of the model against two extreme strategies, “treat all” and “treat none”; a greater net benefit indicated greater clinical practical value.

### 2.7 Validation

Independent single-center clinical ICU data (371 patients, including 17 with MDR and 354 without MDR) were used for external validation to evaluate the generalizability of the optimal model. The validation indicators, including the area under the curve (AUC), accuracy, sensitivity, specificity, F1 score, Brier score, and DCA curves, were consistent with those used for internal validation, ensuring that the model maintained stable predictive performance in different ICU populations. Moreover, descriptive analysis of baseline characteristics was performed for the external validation set, and differences in indicators between the MDR and non-MDR groups were compared to verify the rationality of the baseline data.

### 2.8 Model interpretation and visualization

The model with optimal comprehensive performance was selected. Shapley additive explanations (SHAP) analysis was employed to elucidate the importance of each core feature and its influence direction on MDR risk. On the basis of the optimal model and the five core features, a visualized nomogram was developed to achieve rapid individualized quantification of MDR risk. The nomogram included a feature axis, a scoring axis, and a risk axis, facilitating rapid bedside use by clinicians.

### 2.9 Statistical analysis

**FIGURE 1:**
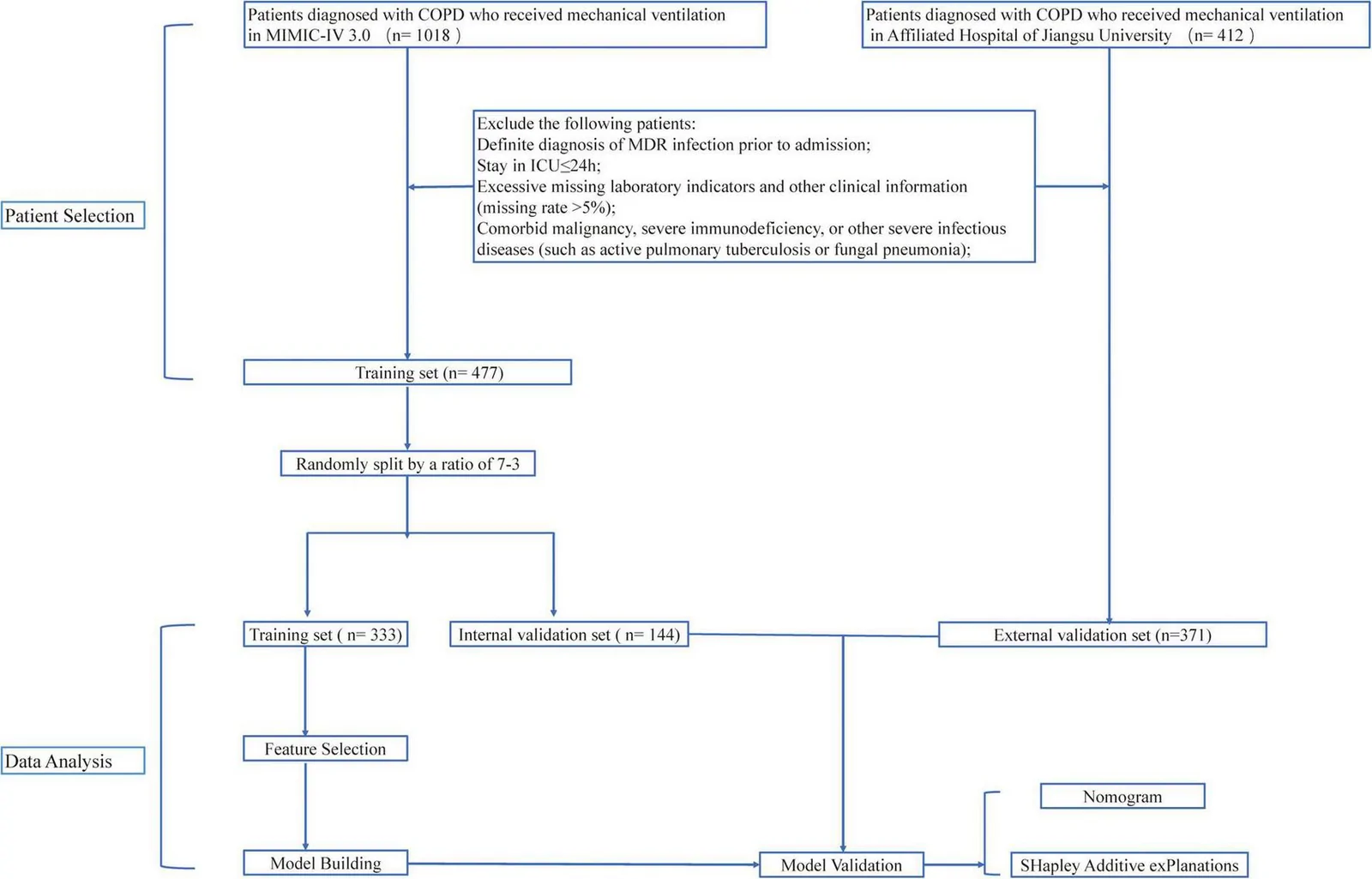
Flowchart of this study.

Patients were grouped according to whether MDR infection occurred in those with COPD receiving mechanical ventilation, and the statistical significance of differences in continuous variables was assessed. Continuous variables with a normal distribution are expressed as the mean ± standard deviation and were analyzed via the *t*-test; variables without a normal distribution are expressed as the interquartile range (IQR) and were analyzed via the Wilcoxon rank-sum test. Categorical variables are expressed as percentages and were compared via the chi-square test. After patients with more than 5% data loss were excluded, multiple imputation was adopted to handle missing data. All selected variables had less than 30% missing values. SMOTE was used to address imbalanced data. Data processing and analysis were performed via R (4.3.0) and Zstats v1.0^1^. A two-sided *p*-value < 0.05 was considered statistically significant. The above research methods and workflow are detailed in Figure 1.

## 3 Results

### 3.1 Baseline characteristics of the study population

A total of 477 ICU patients with COPD receiving mechanical ventilation from the MIMIC database were randomly divided into a training group (*n* = 333) and an internal validation group (*n* = 144) at a 7:3 ratio. A comparison of the data between the two groups revealed that only alanine aminotransferase (ALT), aspartate aminotransferase (AST), and chronic kidney disease (CKD) data had *p*-values < 0.05, whereas the remaining demographic data, laboratory indicators, and comorbidity data were not significantly different (*p* > 0.05). These findings indicated satisfactory randomization effects and comparable baseline characteristics between the two groups of ICU patients, which supported their use for subsequent model construction and validation. The differential analysis of the included influencing factors for the training and internal validation groups is presented in Table 1.

**TABLE 1:** Baseline characteristic comparison between training cohort and validation cohort.

Variables	Total (*n* = 477)	Test (*n* = 144)	Train (*n* = 333)	*P*
Gender, *n* (%)				0.143
Female	233 (48.85)	63 (43.75)	170 (51.05)	–
Male	244 (51.15)	81 (56.25)	163 (48.95)	–
Age, mean ± SD	70.30 ± 10.90	69.94 ± 10.63	70.46 ± 11.04	0.638
IBM, mean ± SD	30.94 ± 10.27	30.57 ± 8.71	31.10 ± 10.88	0.605
Wbc, mean ± SD	12.73 ± 6.97	13.41 ± 7.17	12.43 ± 6.87	0.156
Rbc, mean ± SD	3.73 ± 0.78	3.72 ± 0.76	3.73 ± 0.78	0.840
Platelet count, mean ± SD	212.04 ± 103.58	206.92 ± 92.75	214.25 ± 107.99	0.479
Hemoglobin, mean ± SD	11.06 ± 2.25	11.04 ± 2.29	11.07 ± 2.23	0.909
Rdw, mean ± SD	15.36 ± 2.17	15.46 ± 2.21	15.31 ± 2.15	0.507
Hematocrit, mean ± SD	34.70 ± 7.09	34.55 ± 6.94	34.76 ± 7.16	0.770
Sodium, mean ± SD	138.97 ± 5.43	138.61 ± 5.36	139.13 ± 5.45	0.337
Potassium, mean ± SD	4.47 ± 0.67	4.46 ± 0.68	4.48 ± 0.67	0.742
Calcium total, mean ± SD	8.41 ± 0.72	8.37 ± 0.72	8.42 ± 0.71	0.432
Chloride, mean ± SD	101.38 ± 6.89	101.18 ± 7.09	101.47 ± 6.81	0.667
Glucose, mean ± SD	150.37 ± 50.31	149.08 ± 52.25	150.93 ± 49.52	0.713
Anion gap, mean ± SD	13.71 ± 3.75	13.48 ± 3.79	13.81 ± 3.73	0.388
PH, mean ± SD	7.34 ± 0.08	7.34 ± 0.08	7.34 ± 0.08	0.576
PCO_2_, mean ± SD	54.16 ± 14.67	53.41 ± 14.35	54.49 ± 14.82	0.463
PO_2_, mean ± SD	103.40 ± 58.85	102.21 ± 55.22	103.92 ± 60.43	0.770
Lactate, mean ± SD	1.78 ± 1.22	1.84 ± 1.44	1.76 ± 1.11	0.537
Total CO_2_, mean ± SD	29.73 ± 7.60	29.59 ± 7.85	29.79 ± 7.49	0.793
PT, mean ± SD	15.76 ± 7.27	16.15 ± 7.28	15.59 ± 7.28	0.441
PTT, mean ± SD	39.99 ± 23.09	39.28 ± 22.66	40.30 ± 23.31	0.658
INR, mean ± SD	1.44 ± 0.71	1.49 ± 0.73	1.43 ± 0.70	0.386
ALT, mean ± SD	174.65 ± 552.58	290.29 ± 767.70	124.64 ± 419.07	0.016
AST, mean ± SD	252.51 ± 914.14	445.58 ± 1325.68	169.03 ± 646.51	0.018
Urea nitrogen, mean ± SD	31.55 ± 22.34	32.86 ± 24.22	30.98 ± 21.50	0.401
Creatinine, mean ± SD	1.39 ± 1.11	1.35 ± 1.05	1.40 ± 1.14	0.606
HR, mean ± SD	86.06 ± 16.18	85.51 ± 17.24	86.30 ± 15.73	0.625
Nbpd, mean ± SD	63.65 ± 10.43	64.48 ± 10.71	63.29 ± 10.30	0.255
Nbpm, mean ± SD	76.97 ± 10.11	77.22 ± 10.20	76.87 ± 10.09	0.724
Rr, mean ± SD	20.30 ± 3.69	20.33 ± 3.83	20.28 ± 3.64	0.898
SpO_2_, mean ± SD	95.89 ± 2.37	95.73 ± 2.71	95.96 ± 2.21	0.334
Temperature, mean ± SD	36.89 ± 0.43	36.88 ± 0.45	36.89 ± 0.42	0.828
HT, *n* (%)	213 (44.65)	65 (45.14)	148 (44.44)	0.889
DM, *n* (%)	153 (32.08)	41 (28.47)	112 (33.63)	0.268
HF, *n* (%)	241 (50.52)	72 (50.00)	169 (50.75)	0.880
MI, *n* (%)	47 (9.85)	14 (9.72)	33 (9.91)	0.950
CKD, *n* (%)	98 (20.55)	21 (14.58)	77 (23.12)	0.034
Cirrhosis, *n* (%)	29 (6.08)	9 (6.25)	20 (6.01)	0.918
Hepatitis, *n* (%)	18 (3.77)	5 (3.47)	13 (3.90)	0.820
Pneumonia, *n* (%)	276 (57.86)	87 (60.42)	189 (56.76)	0.457
Stroke, *n* (%)	33 (6.92)	8 (5.56)	25 (7.51)	0.441
HLP, *n* (%)	193 (40.46)	53 (36.81)	140 (42.04)	0.285
AKI, *n* (%)	433 (90.78)	130 (90.28)	303 (90.99)	0.805

IBM, International Business Machines Corporation; Wbc, white blood cell; RBC, red blood cell; Rdw, red cell distribution width; PH, potential of hydrogen; PCO_2_, partial pressure of carbon dioxide; PO_2_, partial pressure of oxygen; PT, prothrombin time; PTT, partial thromboplastin time; INR, international normalized ratio; ALT, alanine aminotransferase; AST, aspartate aminotransferase; HR, heart rate; SPO_2_, saturation of peripheral oxygen; Hospday, length of stay; ARF, acute renal failure; HT, hypertension; DM, diabetes mellitus; HF, heart failure; MI, Myocardial Infarction; HLP, hyperlipidemia; CKD, chronic kidney disease; AKI, acute kidney injury.

### 3.2 Feature selection results

**FIGURE 2:**
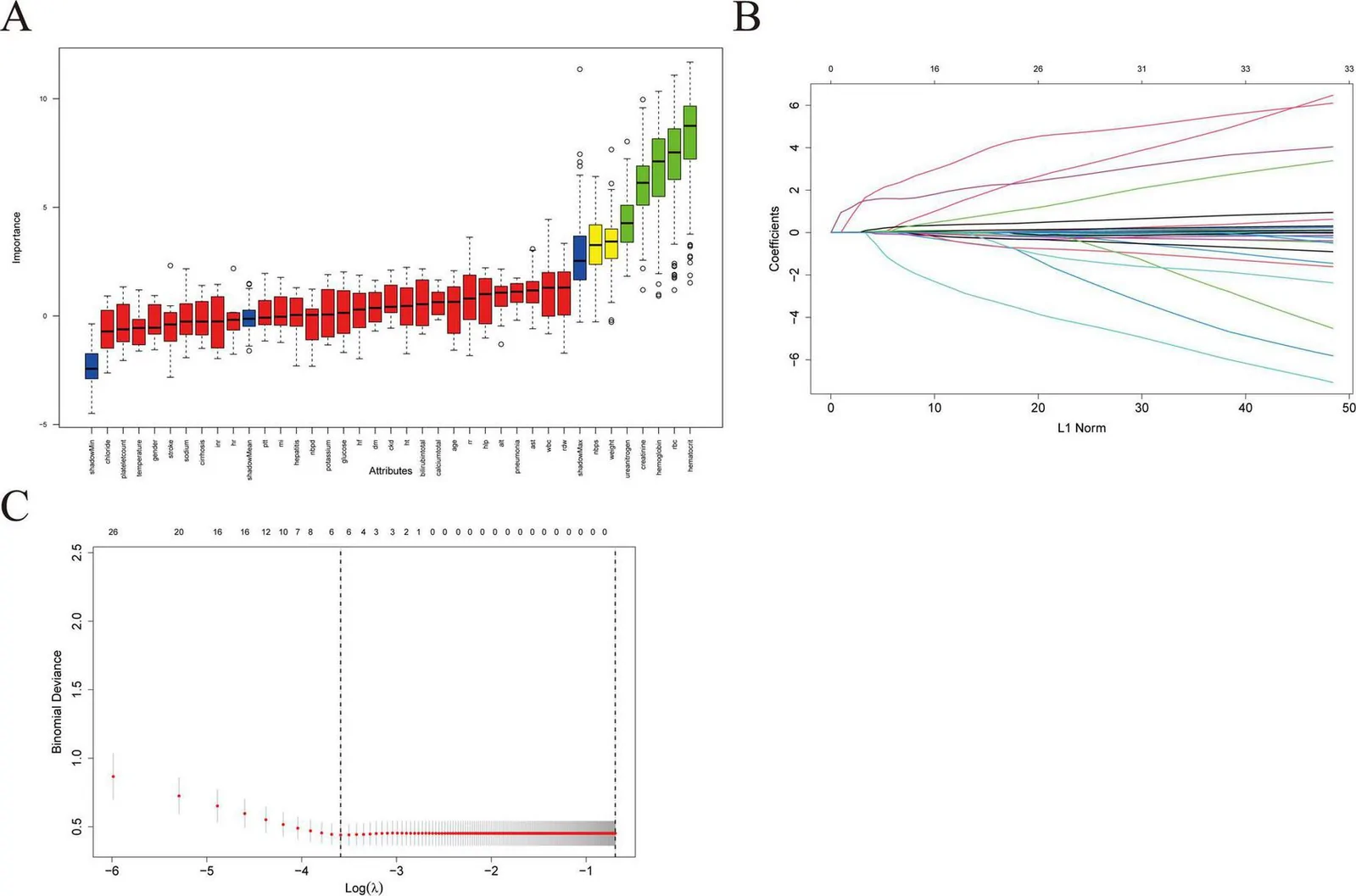
Feature selection with Boruta and LASSO regression. **(A)** The box plot displays the minimum, mean, and maximum shadow scores. Variables with green box plots were identified as important variables, yellow as tentative variables, and red as rejected variables. **(B)** LASSO regression coefficient path plot. **(C)** Cross-validation plot for regression.

Following preliminary screening by the Boruta algorithm, 12 potential predictors associated with MDR infection were retained, as shown in Figure 2A. Further screening through LASSO regression eliminated redundant and low-contribution indicators. Five core predictors were ultimately identified, ranked by model contribution from highest to lowest: hematocrit>creatinine>hemoglobin>red blood cell count (RBC)> lood urea nitrogen (BUN). Among these variables, hematocrit had the greatest contribution (Gain ≈ 0.24) and was identified as the primary variable influencing MDR infection risk, as shown in Figures 2B, C.

### 3.3 Model development and internal validation

**FIGURE 3:**
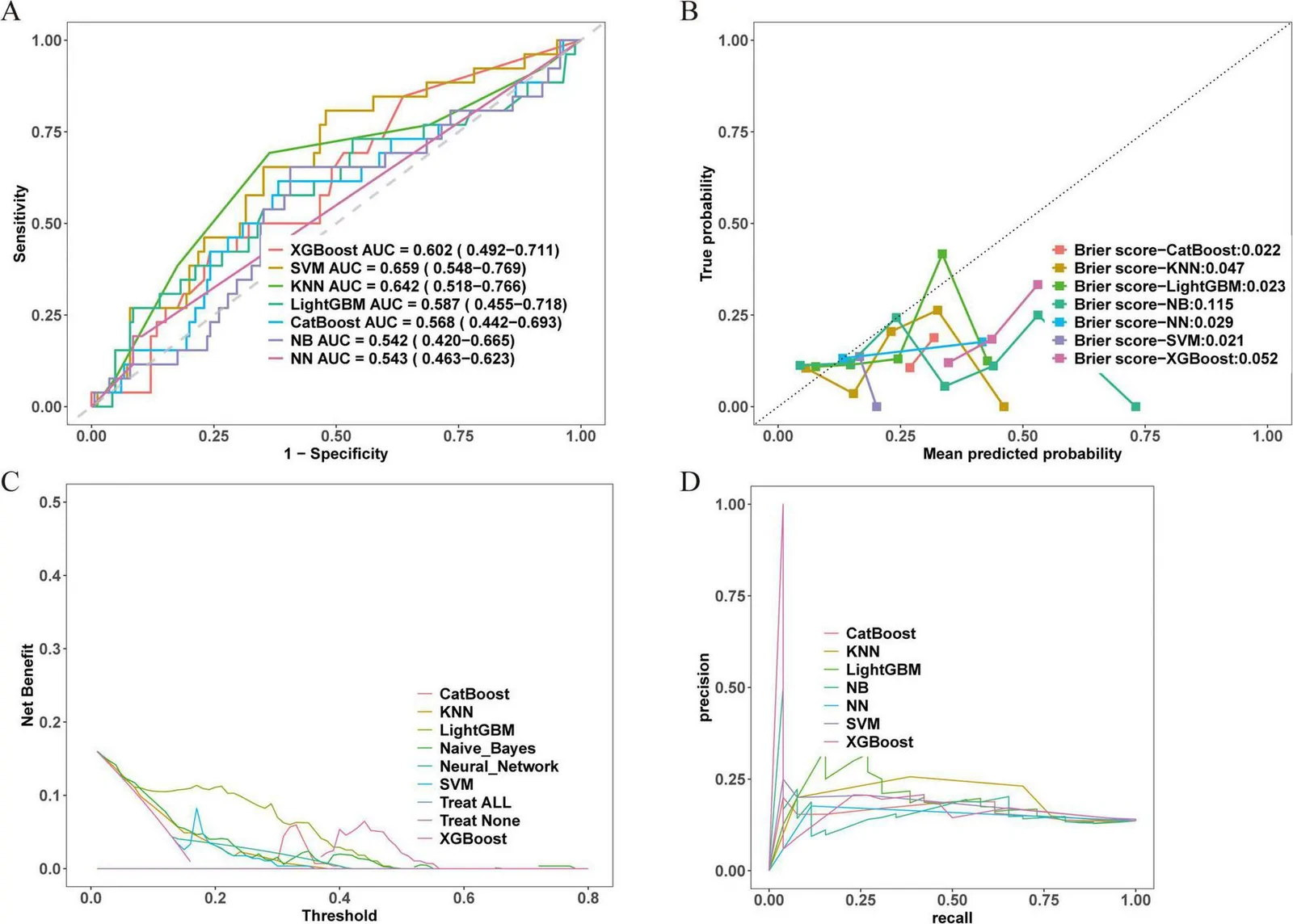
Performance and comparison of seven machine learning prediction models. **(A)** ROC curves for the internal validation set; **(B)** calibration curves for the internal validation set; **(C)** decision curve analysis for the internal validation set; **(D)** recall for the internal validation set.

Seven machine learning models—XGBoost, LightGBM, CatBoost, SVM, KNN, GNB, and NN—were constructed on the basis of the training set (333 ICU patients). After parameter optimization through 10-fold cross-validation, the internal validation results demonstrated that the KNN model achieved optimal comprehensive performance. The complete performance metrics for this model were as follows: AUC = 0.642 (95% CI: 0.518–0.766), accuracy = 0.664, sensitivity = 0.692, specificity = 0.636, F1 score = 0.662, and Brier score = 0.047. The calibration curve indicated good agreement between the predicted probabilities and actual observed probabilities. The complete performance metrics for the SVM model were as follows: AUC = 0.659 (95% CI: 0.537–0.781), accuracy = 0.658, sensitivity = 0.673, specificity = 0.641, F1 score = 0.657, and Brier score = 0.051, with satisfactory calibration curve fitting. The Hosmer-Lemeshow goodness-of-fit test of SVM on internal validation set showed HL statistic = 14.96, degrees of freedom = 8, *P* = 0.06 (Supplementary Table 1), quantitatively confirming acceptable consistency between predicted MDR risks and actual observed events. The core key data (AUC values) for the remaining five models are as follows: XGBoost model AUC = 0.602, LightGBM model AUC = 0.587, CatBoost model AUC = 0.568, GNB model AUC = 0.542, and NN model AUC = 0.543, as shown in Figure 3A and Table 2. By contrast, the three tree ensemble algorithms (XGBoost, LightGBM, CatBoost) exhibited poor calibration via HL test (all *P* < 0.0001, Supplementary Table 1), while KNN and NN showed acceptable HL fitting; we finally selected SVM as the optimal model considering its comprehensive discrimination, calibration and clinical performance. To further clarify the rationale for selecting the KNN model as the optimal model, this study supplemented a multi-dimensional head-to-head comparison between KNN and SVM under three resampling strategies, namely the Synthetic Minority Oversampling Technique (SMOTE), Adaptive Synthetic Sampling (ADASYN), and random undersampling, covering four dimensions: discrimination, calibration, overfitting resistance, and clinical utility. The results showed that although the SVM model achieved a slightly higher area under the curve (AUC) in internal validation (0.659 vs. 0.642 for KNN), the KNN model exhibited a significantly lower degree of overfitting with a smaller performance drop from the training set to the test set. The AUC values of internal and external validation were highly consistent (0.642 vs. 0.639), indicating stronger generalizability of the KNN model across independent cohorts. From the perspective of its clinical positioning as an early preliminary screening tool, the KNN model delivered a substantially higher sensitivity (0.692 vs. 0.462 for SVM), which is of critical importance for reducing the missed diagnosis rate of multidrug-resistant (MDR) bacterial infections. In the imbalanced classification scenario, the KNN model outperformed SVM in F1 score, and generated greater net benefit across most clinically relevant threshold probability ranges in decision curve analysis. Although SVM showed a marginally superior Brier score in calibration assessment, the calibration performance of the KNN model still fell within the acceptable range for clinical risk stratification. Taken together, in line with the clinical demand for rapid bedside preliminary screening, this study selected the KNN model as the optimal model for its better stability, lower missed diagnosis risk, and higher clinical practical value. In addition, we supplemented the variable importance analysis of the SVM model based on Gain values, with the relevant results presented in Supplementary Figure 1. The analysis demonstrated that all five core predictors played effective predictive roles in both models. Creatinine and hematocrit consistently ranked in the top two positions in both models, acting as core variables driving MDR risk prediction. The discrepancy between the two models lay in the ranking of the top predictor: hematocrit made the highest contribution in the KNN model, while creatinine ranked first in the SVM model. Further analysis confirmed that the direction of influence of each core predictor on MDR infection risk was completely consistent between the two models, verifying that the screened core predictors possess cross-algorithm robustness. Decision curve analysis (DCA) revealed that within the threshold probability range of 0.1–0.5, the net benefit of the KNN model was significantly greater than that of the other six models and the two extreme strategies of “treat all” and “treat none,” as shown in Figure 3B. The net benefit of the SVM model was second only to that of the KNN model, suggesting that both models possessed high clinical utility, with the KNN model demonstrating optimal performance, as shown in Figure 3C. Figure 3D showed the precision recall curves of seven predictive models.

**TABLE 2:** Performance of machine-learning models for predicting MDR.

Model	Set	AUC	Sensitivity	Specificity	PPV	NPV	Precision	Recall	F1	Accuracy
XGBoost	Train	0.850	0.812	0.811	0.464	0.955	0.464	0.812	0.591	0.812
XGBoost	Test	0.602	0.385	0.77	0.208	0.888	0.208	0.385	0.27	0.577
CatBoost	Train	0.834	0.771	0.773	0.407	0.944	0.407	0.771	0.532	0.772
CatBoost	Test	0.568	0.423	0.739	0.204	0.891	0.204	0.423	0.275	0.581
SVM	Train	0.842	0.708	0.941	0.708	0.941	0.708	0.708	0.708	0.825
SVM	Test	0.659	0.462	0.721	0.207	0.895	0.207	0.462	0.286	0.591
KNN	Train	0.684	0.625	0.647	0.263	0.895	0.263	0.625	0.37	0.636
KNN	Test	0.642	0.692	0.636	0.231	0.929	0.231	0.692	0.346	0.664
LightGBM	Train	0.927	0.854	0.874	0.577	0.967	0.577	0.854	0.689	0.864
LightGBM	Test	0.587	0.423	0.721	0.193	0.888	0.193	0.423	0.265	0.572
NB	Train	0.731	0.667	0.702	0.311	0.913	0.311	0.667	0.424	0.684
NB	Test	0.542	0.346	0.703	0.155	0.872	0.155	0.346	0.214	0.525
NN	Train	0.619	0.354	0.882	0.378	0.871	0.378	0.354	0.366	0.618
NN	Test	0.543	0.192	0.897	0.227	0.876	0.227	0.192	0.208	0.545

XGBoost, eXtreme Gradient Boosting; SVM, support vector machine; LightGBM, light gradient boosting machine; CatBoost, categorical boosting; NB, naive bayes; NN, neural network.

### 3.4 External validation of the model

**FIGURE 4:**
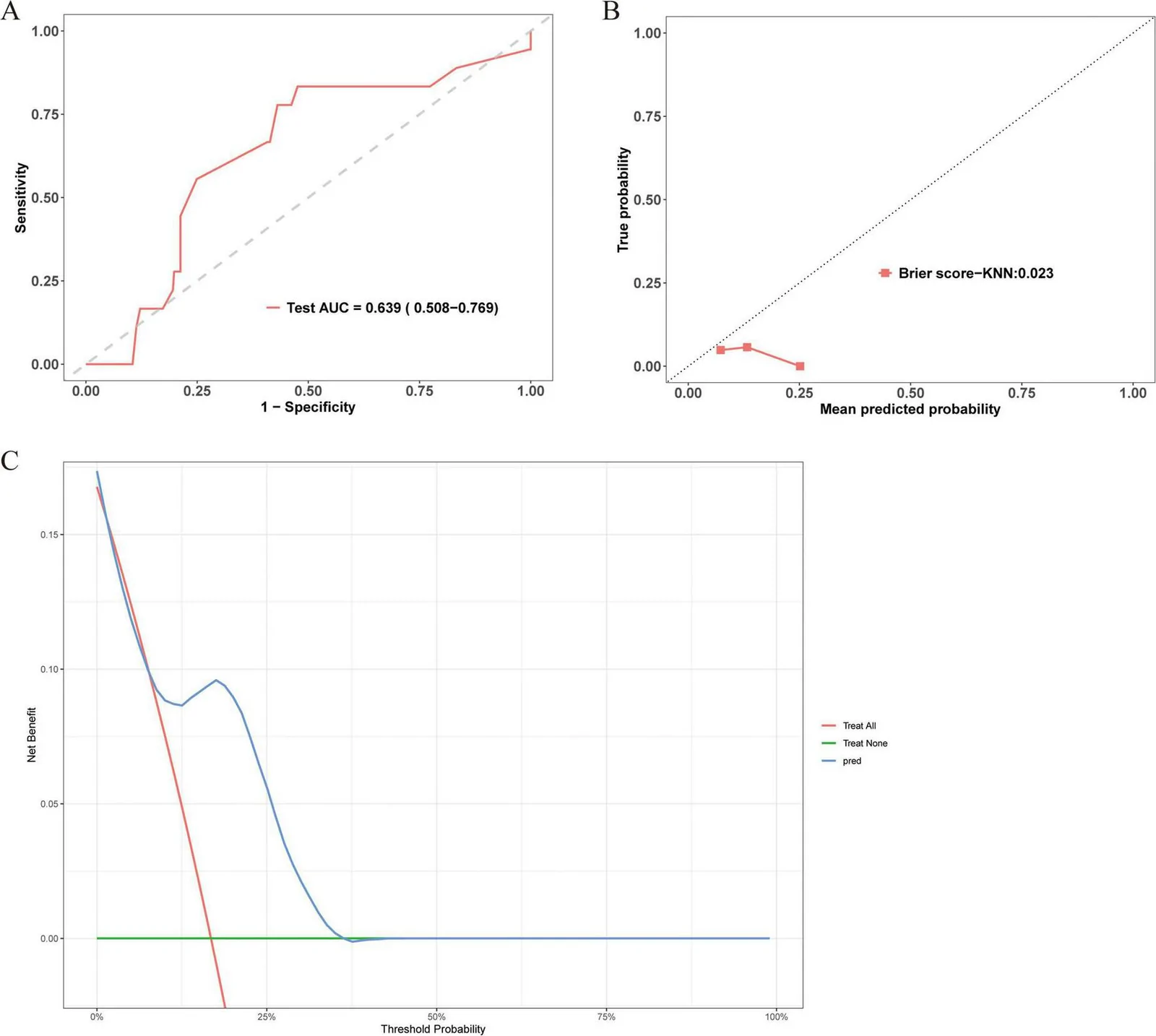
External validation of the K-nearest neighbor (KNN) model’s predictive performance. **(A)** ROC curve for the external validation set; **(B)** calibration curve for the external validation set; **(C)** decision curve analysis for the external validation set.

The external validation set included a total of 371 ICU patients with COPD receiving mechanical ventilation, among whom 17 (4.58%) developed MDR infection and 354 (95.42%) did not, as detailed in Table 3. External validation of the optimal KNN model was performed in conjunction with the baseline characteristics of the external validation set (Table 3). The age of the ICU patients in this validation set was 76.42 ± 7.69 years, and the sex distribution was 326 males (87.87%) and 45 females (12.13%). The validation results showed that the KNN model external validation AUC was 0.639 (95% CI: 0.508–0.769), which was highly consistent with the internal validation AUC (0.642), suggesting the stable discriminative ability of the model. Accuracy = 0.652, sensitivity = 0.647, specificity = 0.653, and F1 score = 0.650. These discrimination metrics were essentially consistent with the internal validation results, indicating that the model maintained satisfactory predictive accuracy in different ICU populations, as shown in Figure 4A. The Brier score was 0.023, which was slightly different from the internal validation Brier score (0.047). The calibration curve demonstrated good agreement between the predicted probabilities and actual observed probabilities for ICU patients in the external validation set, with a small prediction deviation, as shown in Figure 4B. The HL test for SVM on external validation set returned HL statistic = 14.03, df = 8, *P* = 0.081 (Supplementary Table 1), further verifying stable and favorable calibration performance of the SVM model in independent clinical cohorts. Decision curve analysis (DCA) revealed that across the full threshold probability range of 0%–100%, the net benefit of the KNN model was significantly greater than that of the “treat all” and “treat none” strategies. The net benefit peaked in the 25%–75% moderate-to-high risk threshold segment. The MDR incidence rate in the external validation set (4.58%) suggested that the model could effectively screen moderate- to high-risk patients in clinical practice, avoiding both overintervention and underintervention, as shown in Figure 4C.

**TABLE 3:** Baseline characteristics of the external validation set.

Variables	Total (*n* = 371)	No-MDR (*n* = 354)	MDR (*n* = 17)	*P*
Age, mean ± SD	76.42 ± 7.69	76.51 ± 7.77	74.71 ± 5.61	0.347
Gender, *n* (%)				0.669
Female	45 (12.13)	44 (12.43)	1 (5.88)	–
Male	326 (87.87)	310 (87.57)	16 (94.12)	–
RBC, mean ± SD	4.31 ± 0.63	4.31 ± 0.62	4.21 ± 0.85	0.517
Hemoglobin, mean ± SD	130.40 ± 19.81	130.52 ± 19.54	128.00 ± 25.50	0.609
Hematocrit, mean ± SD	40.46 ± 6.16	40.49 ± 6.09	39.86 ± 7.73	0.682
Urea nitrogen, mean ± SD	7.06 ± 3.22	7.00 ± 3.11	8.41 ± 4.99	0.280
Creatinine, mean ± D	76.82 ± 33.08	77.06 ± 33.04	71.42 ± 34.79	0.506

RBC, red blood cell.

### 3.5 SHAP interpretability analysis

**FIGURE 5:**
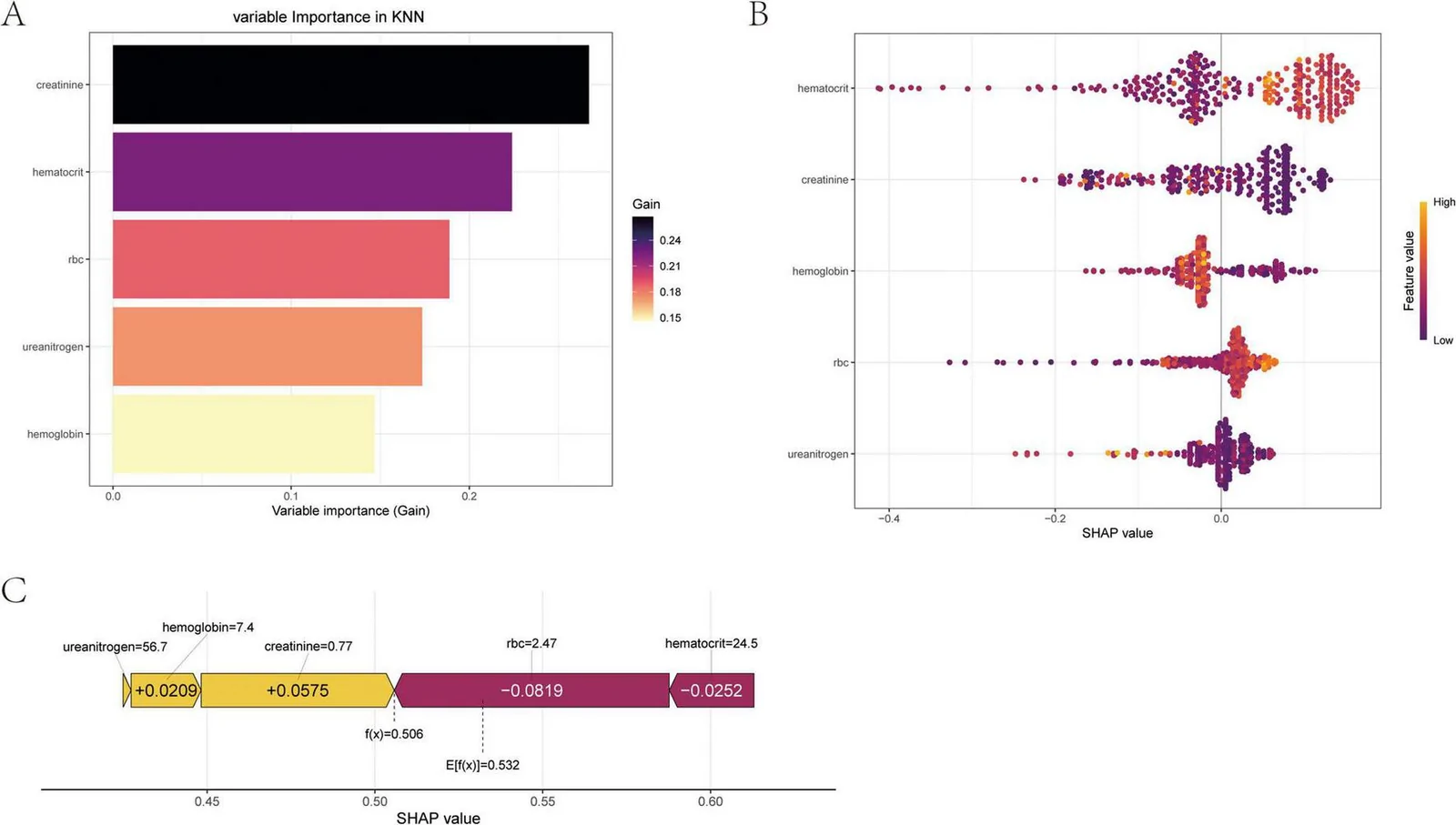
Interpretability analysis of the K-nearest neighbor (KNN) model. **(A)** Shapley additive explanations (SHAP) summary plot of features of the KNN model. **(B)** SHAP dependence plots of features. **(C)** Interpretability analysis of one individual sample.

Shapley additive explanations analysis was performed on the KNN model. The results revealed that the feature importance ranking based on gain was hematocrit>creatinine>hemoglobin>RBC>BUN. The hematocrit demonstrated the greatest contribution and was identified as the primary predictor influencing MDR infection risk, as shown in Figure 5A. The SHAP scatter plot revealed clear influence directions of the core features on MDR infection risk: when hematocrit, RBC, and hemoglobin levels increased, SHAP values were positive, suggesting elevated MDR infection risk in ICU patients with COPD receiving mechanical ventilation; when BUN and creatinine levels increased, SHAP values were negative, suggesting decreased MDR infection risk in this ICU patient population. The gradient relationship between feature values from low to high and SHAP values was evident, indicating that these indicators could quantitatively drive MDR risk prediction. This pattern was also reflected in the external validation set and was consistent with the distribution trends of core indicators in the baseline characteristics of ICU patients in the external validation set, as shown in Figure 5B. Moreover, individualized attribution analysis was performed for one typical ICU patient with COPD who was receiving mechanical ventilation. The base prediction value of the model was E[f(x)] = 0.532, and after correction for each core feature, the final MDR risk value was f(x) = 0.506. The protective factors (decreasing MDR risk) included hematocrit = 24.5 (SHAP = −0.0252) and RBC = 2.47 (SHAP = −0.0819). The risk factors (increasing MDR risk) included BUN = 56.7 (SHAP = +0.0209) and hemoglobin = 7.4 (SHAP = +0.0575). A creatinine value of 0.77 had no significant influence on the final prediction result. These findings achieved full-chain interpretability from population-level patterns to individual-level decisions, providing objective evidence for individualized risk assessment of ICU patients, as shown in Figure 5C.

### 3.6 Nomogram construction and validation

**FIGURE 6:**
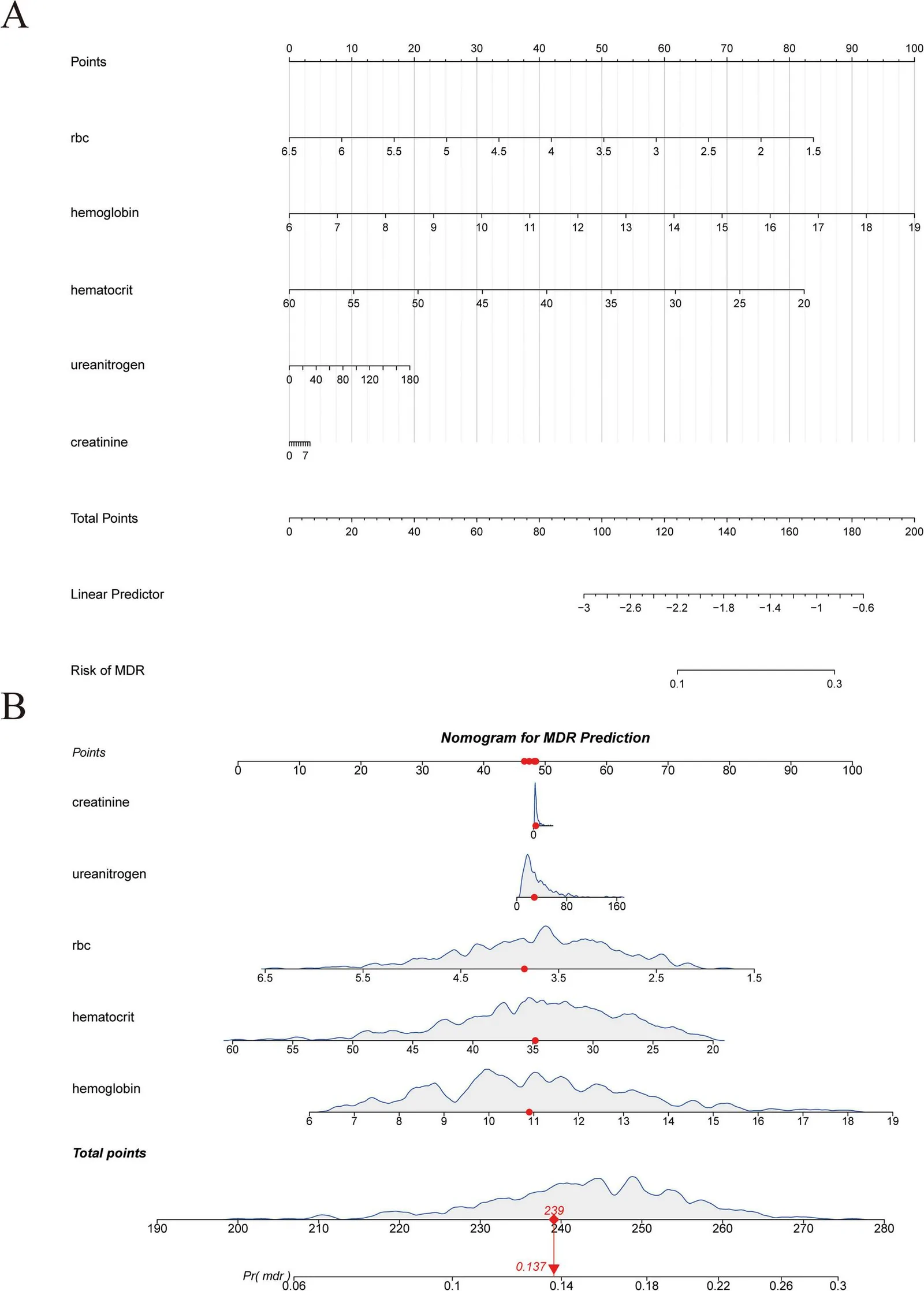
Construction of two different nomograms. **(A)** Nomogram with five scenarios. **(B)** Interactive nomogram.

Two nomograms were constructed in this study. Figure 6A integrates five important predictive variables—RBC, hemoglobin, hematocrit, BUN, and creatinine—to intuitively assess the risk of concurrent MDR infection in ICU patients with COPD receiving mechanical ventilation. The figure clearly presents the scoring scales for each predictive variable, the total score calculation method, and the corresponding MDR infection risk range. Figure 6B displays an interactive nomogram. For the example patient, the score was 239 points, corresponding to an MDR infection probability of approximately 13.7%, providing a rapid and easily interpretable method for individual risk assessment that facilitates rapid bedside application by clinicians.

## 4 Discussion

This study was based on the MIMIC-IV database and single-center clinical data and successfully constructed and validated a risk prediction model for MDR infection in ICU patients with COPD receiving mechanical ventilation. The core findings and clinical significance were as follows:

First, through joint screening via the Boruta algorithm and LASSO regression, this study identified five core predictors: RBC, hemoglobin, hematocrit, BUN, and creatinine. All of these indicators are routine laboratory indicators that require no additional testing, can be rapidly obtained, and are cost effective, making them suitable for widespread application in ICUs and intensive care units at primary-level hospitals. Among these parameters, hematocrit served as the primary predictor. Its elevated level suggested that ICU patients might have hemoconcentration and aggravated hypoxia, and the hypoxic state could further impair airway mucosal barrier function, promoting colonization and infection by resistant organisms. This finding was consistent with previous conclusions that “anemia-related indicators are closely associated with drug resistance in pulmonary infections.” (10) Multiple observational studies have consistently demonstrated that chronic kidney disease is an independent risk factor for multidrug-resistant organism urinary tract infection (OR = 1.78, 95% CI 1.15–2.75) (11). The prevalence of multidrug-resistant (MDR) infection reaches 64.7% among patients with CKD (12), and the uremic milieu causes extensive impairment of innate and adaptive immune function. This study revealed that blood urea nitrogen and serum creatinine acted as protective factors. Mechanistically, elevated urea and creatinine may reduce the risk of multidrug-resistant organism infection via the following pathways: urea can disrupt the extracellular polymeric matrix of bacterial biofilms *in vitro* (13, 14), upregulate the expression of antimicrobial peptides LL-37 and β-defensin-2 in cutaneous keratinocytes (15), and urea-driven protein carbamylation may alter the conformation of bacterial functional proteins (16); creatinine downregulates the expression of TLR-2/3/4/7 in macrophages (17). In addition, uremic toxins such as hippuric acid inhibit host multidrug resistance transporters MRP4 and BCRP at clinically relevant concentrations (18), which may indirectly alter the *in vivo* distribution of antibiotics. Baseline data of ICU patients in the external validation set showed no statistically significant differences in the five core indicators between the multidrug-resistant organism infection group and the non-infection group (all *P* > 0.05). This further confirms that the above indicators are independent predictors of multidrug-resistant organism infection rather than baseline confounding factors, improving the reliability of the model.

Second, among the seven machine learning models constructed in this study, the KNN model demonstrated optimal comprehensive performance, with an internal validation AUC of 0.642 and an external validation AUC of 0.639. The close agreement between these values, combined with low Brier scores and prominent net benefit in DCA, indicated that this model possessed favorable discrimination, calibration, and generalizability for predicting MDR infection risk in ICU patients with COPD receiving mechanical ventilation, without obvious overfitting. Although the internal validation AUC of the SVM model (0.659) was slightly higher than that of the KNN model and its performance metrics were comparable to those of the KNN model, this study ultimately selected the KNN model as the optimal model. The core reasons are the significant differences between the two in terms of clinical applicability, interpretability, and generalizability. First, the KNN model has a simple structure that is easy to understand and operate without requiring a complex parameter tuning process. ICU clinicians and healthcare staff at primary-level medical institutions could rapidly apply it in clinical practice without mastering profound machine learning expertise. In contrast, the SVM model is highly dependent on parameter settings (such as kernel function selection and penalty coefficient adjustment), and the parameter optimization process is cumbersome. Different parameter combinations could significantly affect model predictive performance, which is unfavorable for routine clinical promotion (19). Second, the KNN model offered superior interpretability. SHAP analysis clearly elucidated the influence direction and magnitude of each core feature on MDR infection risk, providing an explicit decision-making basis for clinical intervention. Supplementary SHAP analysis of the SVM model also showed consistent feature importance and influence direction, but the overall interpretability of the SVM model is still weaker than that of KNN due to its own algorithm characteristics. The SVM model, however, belongs to the “black box” category, making it difficult to intuitively explain the logic underlying the prediction results. This method could not adequately clarify the associations between clinical indicators and MDR infection, which was unfavorable for clinical healthcare staff to understand and trust the model predictions (20). Third, the KNN model showed lower sensitivity to sample distribution. Under three different resampling strategies, the AUC fluctuation range of the KNN model was less than 0.02, while the SVM model had a fluctuation range of more than 0.04. In this study, where the external validation set had a low MDR incidence rate (4.58%) and imbalanced sample distribution, it still maintained stable predictive performance. In contrast, the SVM model is prone to decreased prediction stability and less reliable generalizability when faced with imbalanced samples and noisy data (21). Compared with complex algorithms such as XGBoost and LightGBM, the KNN model similarly possesses the advantages of a simple structure and easy promotion without requiring complex parameter tuning. It is more suitable for rapid application by ICU clinicians, especially for MDR risk screening in intensive care units at primary-level medical institutions (22). Furthermore, the MDR incidence rate in ICU patients in the external validation set was 4.58%, representing a low-incidence population. The fact that the KNN model still maintained stable predictive performance under these circumstances further demonstrated its clinical practicality (23).

Third, the application of SHAP analysis broke through the “black box” dilemma of machine learning models, clarifying the importance of each core feature and its influence direction on MDR risk in ICU patients. This study not only validated the clinical significance of the five predictors but also provided specific evidence for individualized intervention in ICU patients through individual case attribution analysis. For example, for ICU patients with low hematocrit and red blood cell (RBC) levels, measures such as improving oxygenation and correcting anemia could be implemented to reduce MDR infection risk. For ICU patients with abnormally elevated BUN and creatinine levels, attention should be given to their renal function status, avoiding excessive use of nephrotoxic antimicrobial agents while optimizing prevention and control strategies by leveraging their protective effects. This pattern of effects was confirmed in the external validation set, indicating that the interpretability of the model possessed good generalizability (24, 25).

Additionally, the internal and external validation AUC values of the KNN model constructed in this study were 0.642 and 0.639, respectively, both of which were maintained at approximately 0.65. These values were consistent with previous studies on MDR infection prediction in patients with COPD and fell within a reasonable range. Reviewing relevant studies, Zhou et al. constructed a prediction model for MDR infection during mechanical ventilation in COPD patients, with internal and external validation AUC values of 0.628 and 0.635, respectively, which were highly consistent with the results of this study (26). Güler et al. employed the random forest algorithm to construct a prediction model with an AUC value of 0.661, which was slightly higher than that of this study. The main reason was that they included 10 predictors, covering additional indicators such as sputum culture results and antimicrobial use history, whereas this study only used five routine laboratory indicators. Although there was a slight difference in predictive performance, the latter offered greater clinical convenience and generalizability (27). Other studies that employed traditional logistic regression to construct models mostly reported AUC values in the range of 0.60–0.68. The AUC value in this study fell within the middle of this range, indicating that the KNN-based model demonstrated superior predictive performance compared with some traditional models and was comparable to similar machine learning models (28). Taken together, although the AUC values in this study did not reach a high discrimination level above 0.70, considering the complexity of MDR infection, the sample size, and the limitations of the predictors in this study, these values were sufficient to meet the needs for preliminary screening of high-risk MDR populations in clinical practice. It should be clearly clarified that this model is only applicable to preliminary risk stratification at the early stage of ICU admission, and cannot replace microbial culture and drug susceptibility testing as the gold standard for definitive diagnosis of MDR infection. The clinical value of this model lies in providing auxiliary reference for early clinical decision-making, rather than guiding definitive anti-infection treatment independently. Combined with five easily obtainable routine indicators, the model was more suitable for rapid bedside application in ICUs, embodying the research characteristic of “balancing simplicity and performance” (29).

Finally, the nomogram constructed on the basis of the KNN model transformed the complex machine learning algorithm into a visualized scoring tool. ICU clinicians could complete individualized MDR risk assessments for patients within one minute without mastering complex algorithmic knowledge, significantly improving the clinical practicality and operability of the model. The validation results of high calibration for the nomogram further ensured the accuracy of risk prediction, providing a reliable tool for stratified management of ICU patients. For high-risk ICU patients, broad-spectrum antimicrobial agents could be initiated early, with enhanced airway care and isolation protection. For low-risk ICU patients, antimicrobial misuse could be avoided, reducing the emergence of drug resistance and achieving precision anti-infection prevention and control. Preliminary testing of the nomogram applied to ICU patients in the external validation set revealed that its predictive effect was consistent with that of the model and that it was applicable to bedside assessment in different ICU populations (30).

This study had certain limitations. First, the retrospective design might have introduced selection bias (31). Second, the external validation used single-center ICU data (371 patients, including 17 with MDR), with a relatively small sample size and low MDR incidence rate. The generalizability of the model to multicenter ICU populations from different regions requires further validation (32). Third, as a major limitation of this study, universally recognized clinical predictors associated with MDR infection were not included in the model, including mechanical ventilation duration, pre-ICU antibiotic exposure history, tracheotomy status, glucocorticoid use, and sputum microbiological results. The absence of these clinically meaningful covariates was mainly restricted by data integrity and recording consistency between the two cohorts, which may limit the discriminatory ability of the model to a certain extent. These factors will be prioritized for supplementation in future prospective studies to further optimize model performance (33). Furthermore, there is still room for improvement in the discriminatory performance of the KNN model. Systematic optimization plans will be carried out in subsequent research: Expanding the sample size and conducting multicenter prospective validation to reduce selection bias caused by retrospective design and further improve model generalizability; Enriching the dimension of predictor variables, incorporating the above-mentioned clinical risk factors and microbiological data to enhance the model’s discriminatory ability; Optimizing algorithm frameworks and feature engineering strategies under multiple resampling methods to further balance model performance and clinical practicability (34).

Future research could include multicenter prospective studies, incorporate more potential predictors for ICU patients, and optimize the model structure. Moreover, the nomogram could be transformed into a mobile application or web-based tool to further enhance its clinical usability, providing stronger support for early warning and precision prevention and control of MDR infection in ICU patients with COPD receiving mechanical ventilation (35).

## 5 Conclusion

The KNN model and visualized nomogram constructed via machine learning in this study, with RBC, hemoglobin, hematocrit, BUN, and creatinine as core predictors, could stably and accurately predict the risk of MDR infection in ICU patients with COPD receiving mechanical ventilation. The model demonstrated favorable discrimination, calibration, and clinical practicality, with simple operation and low cost. External validation further confirmed the stable generalizability of the model, making it suitable for preliminary early screening and individualized management of high-risk MDR populations in ICUs and intensive care units at primary-level hospitals, providing auxiliary reference for clinical anti-infection decision-making and reducing the transmission of resistant organisms. It should be emphasized that this model cannot replace microbial culture and drug susceptibility testing as the gold standard for definitive diagnosis.

## Data Availability

The original contributions presented in this study are included in this article/Supplementary material, further inquiries can be directed to the corresponding authors.

## Footnotes

1
www.zstats.net

## Supplementary material

Supplementary Figure 1 Shapley additive explanations (SHAP) interpretability analysis of the support vector machine (SVM) model. **(A)** Feature importance ranking. **(B)** SHAP summary plot of features.
