# Supplementary material for: Development and internal-external validation of a machine learning-based risk prediction model for multidrug resistance in mechanically ventilated ICU patients with chronic obstructive pulmonary disease
Source: Front Med (Lausanne). 2026 Aug 20;13:1899252. doi: 10.3389/fmed.2026.1899252 (PMC13538846; doi:10.3389/fmed.2026.1899252)
Supplement: Supplementary file 1 [file Table_1.docx]

**Supplementary Table 1**. Summary of Hosmer-Lemeshow Test Results for Six Machine Learning Models

| Model | Dataset | HL Statistic | Degrees of Freedom | P-value | Goodness of Fit |
| --- | --- | --- | --- | --- | --- |
| XGBoost | Internal Validation Set | 64.94 | 8 | <0.0001 | Poor |
| LightGBM | Internal Validation Set | 40.79 | 8 | <0.0001 | Poor |
| CatBoost | Internal Validation Set | 33.47 | 8 | 0.0001 | Poor |
| SVM | Internal Validation Set | 14.96 | 8 | 0.06 | Good |
| KNN | Internal Validation Set | 2.78 | 3 | 0.4268 | Good |
| NN | Internal Validation Set | 12.31 | 8 | 0.1378 | Good |
| SVM | External Validation Set | 14.03 | 8 | 0.081 | Good |
